# Relationship between learners’ L2 Motivational Self System and parental investment behavior in learners’ English learning

**DOI:** 10.3389/fpsyg.2024.1380346

**Published:** 2024-05-20

**Authors:** Yu Zhang, Xiaobing Lu, Honggang Liu

**Affiliations:** ^1^Gongping Middle School of Changchun, Changchun, China; ^2^Department of Applied Foreign Language Studies, Nanjing University, Nanjing, China; ^3^School of Foreign Languages, Soochow University, Suzhou, China

**Keywords:** correlation, L2 Motivational Self System, parental investment behavior, senior high school students, English learning

## Abstract

Students’ motivation and learning behavior are significantly impacted by parents’ participation and investment. It has been demonstrated that parental investment behavior could exert a direct effect on students’ L2 Motivational Self System (L2MSS) mediated by parental investment belief. Nevertheless, the relationship between components of parental investment behavior and students’ language learning motivation remains a topic necessitating further scholarly investigation. In response to this gap, we conducted a quantitative study involving a survey of 900 high school students to explore the relationship between students’ English learning motivation, as conceptualized by the L2 Motivational Self System and parental investment behavior based on a four-component model. The findings in this study indicated that high school students exhibited moderate levels of L2MSS and relatively low levels of parental investment behavior. Moreover, students’ L2MSS was found to have a significant positive correlation with the global parental investment behavior, with parental emotional investment behavior emerging as a positive predictor of high school students’ L2MSS. These results underscore the importance of parental provision of sufficient economic, relationship, knowledge, and emotional support in cultivating a nurturing and supportive familial context conducive to the development of students’ positive future selves.

## 1 Introduction

Parents’ active involvement and substantial investment within the family context serve as crucial factors that can significantly affect students’ academic engagement (Fan and Williams, 2010; Castro et al., 2015; Hosseinpour et al., 2015), enhance students’ self-regulation capabilities (Gonzalez-DeHass et al., 2005), support students’ goal-setting processes (He et al., 2015), and improve students’ academic outcomes (Fan and Chen, 2001). In the Chinese EFL (English as a Foreign Language) educational framework, English is a required component of the senior high school curriculum. Parents demonstrate a commitment to investing financial and material resources in their children’s language education to enhance future career prospects. Parents’ perceptions of the importance and necessity of English learning significantly shape students’ motivational levels.

Parental investment behavior was found to directly influence students’ English learning motivation (Liu, 2024). Research on the interplay between parental investment behavior and students’ English learning motivation warrants further exploration, as it aligns with the burgeoning focus on learner-internal factors within external contexts. This study aims to examine the relationship between parental investment behavior and students’ L2MSS to achieve a more nuanced understanding of how various forms of parental investment behavior—including economic, knowledge-based, relational, and emotional—impact students’ motivation to learn English.

## 2 Literature review

### 2.1 L2 Motivational Self System

Motivation has been a prominent research topic due to its key role in supporting long-term acquisition (Dörnyei and Ryan, 2015; Csizér, 2019; Al-Hoorie and Szabó, 2022; Yousefi and Mahmoodi, 2022). Dörnyei (2005) introduced the L2 Motivational Self System consisting of three components: the ideal L2 self, the ought-to L2 self and the L2 learning experience. The ideal L2 self represents the aspirational self-concept that second language learners aim to embody in the future. The ought-to L2 self encompasses the perceived obligation to meet external expectations and avert negative outcomes. Finally, the L2 learning experience pertains to learners’ educational experiences, influenced by pedagogical contexts such as instructors, peers, curriculum, and personal achievements (Dörnyei, 2005, 2009).

Subsequently, the L2MSS won a popularity in researching L2 motivation across diverse social-cultural contexts, owing to its adaptability and its capacity to integrate a multiplicity of theoretical perspectives (Al-Hoorie, 2018). A growing interest in motivation has emerged with two major topics. Some studies specifically validated the L2MSS in different contexts, such as China (You and Dörnyei, 2016) and Iran (Papi, 2010) and confirmed the validity of the anticipated construct. Lee and Lee (2020) investigated the L2MSS of 105 college students and 112 high school students in South Korea and found that the level of the ought-to L2 self of high school students was higher than that of university students because compared with university students, the English learning of high school students was mainly motivated to meet the expectations and recognition of others, such as family, teachers and peers.

Several scholarly investigations have examined the association between the L2MSS and other learner-internal and external factors, such as anxiety (Papi, 2010), willingness to communicate (Lee and Lee, 2020), engagement (Zhu et al., 2022), L2 achievement (Moskovsky et al., 2016) and learning style (Kim and Kim, 2014). Research by Magid (2009) revealed distinctions in the conceptualization of the ideal L2 self between middle school and college students. College students’ conceptualization of the ideal L2 self was found to be more intricate and well-developed than that of middle school students, who appeared to struggle with formulating a cohesive and mature vision. College students not only experienced intrinsic satisfaction in learning English but also placed significant emphasis on the practical application of the language for future career advancement.

According to Papi (2010), there is a tendency for students to experience increased levels of anxiety when their learning behavior is heavily influenced by their ought-to L2 self. The external factors that influence students’ L2 learning motivation included students’ family background and learning contexts. Ryan (2009) explored the English learning motivation of 2,397 Japanese middle school students and college students through a mixed-methods study and found that students could gain a higher social status and positive learning experience through proficient English. Khany and Amiri (2018) demonstrated that the ideal L2 self and the L2 learning experience of Iranian high school students were the main motivational factors to stimulate learners’ learning behavior. Li (2019) explored the English learning motivation of 128 Chinese high school students within the framework of L2MSS. The results showed that high school students’ English learning was more inclined to be influenced by language learning experience, and the correlation between English learning experience and expected effort was the highest compared with the ideal L2 self and the ought-to L2 self.

English education constitutes a comprehensive and multifaceted endeavor that encompasses a wide array of elements, including the development of educational policies and curriculum standards, the design of teaching materials, and the establishment of examination and evaluation criteria at the macro level (Liu, 2017). At the micro level, it involves the support of teachers (Liu and Li, 2023) and the financial and emotional investment of parents (Zhang and Liu, 2022). Given this complex structure, it is essential to consider the impact of parental influence within the family environment on students’ motivation to learn a language. Understanding the role of parents in shaping students’ language learning experiences can contribute to the development of more effective strategies to enhance students’ motivation and engagement.

### 2.2 Parental investment behavior

Parents’ involvement in education and educational psychology research has attracted scholarly attention for its significant impact on their children’s academic and emotional development (Boonk et al., 2018). Parental involvement is an essential factor in enhancing students’ academic development (Wigfield et al., 2006; Asgari and Mustapha, 2011; Huang, 2013), promoting students’ self-regulation ability (Gonzalez-DeHass et al., 2005), and improving students’ academic performance (Fan and Chen, 2001). In the language education research, Liu (2017) developed the concept of “parental investment behavior” underpinned by Bourdieu (1986)‘s Capital Theory and the concept of “investment” (Peirce, 1995). The parental investment behavior refers to “forms of investment in their children’s English learning that are commensurate with the parents’ capital in multiple ways and to diverse extents” (Liu, 2024, p.2). The parental investment behavior is classified into economic, relationship, knowledge, and emotional investment behaviors (Liu, 2017, 2024).

According to Liu (2017, 2024), the economic investment behavior pertains to the materials and resources parents provide for their children based on their economic capital, such as enrolling them in English extracurricular activities. The relationship investment behavior involves parents utilizing their social capital to help their children gain access to a key school with higher quality teaching. The knowledge investment behavior is linked to parents’ symbolic and cultural capital, including tutoring their children in language knowledge and discussing foreign language cultural backgrounds at home. The emotional investment behavior relates to parents leveraging their emotional capital, such as offering encouragement and support when their children struggle with academic performance. Parental investment behavior is primarily influenced by factors such as parental investment beliefs, social class, and educational level.

The concept of parental investment behavior aligns with the notion of parental involvement in the broader field of general education; however, parental investment behavior is more specifically grounded in Bourdieu’s (1986) Capital Theory. This framework has been adapted and applied in the context of foreign language education, offering a more targeted approach to understanding the nuances of parental engagement in this specialized domain. Moreover, it covers wider range of parental investment behavior, which enriched and extended the concept of parental involvement. Parental investment behavior encompasses not only specific parental involvement practices such as tutoring children at home (Epstein et al., 2002), participating in school activities (Grolnick and Slowiaczek, 1994), and providing emotional support (Allatt, 1993), but also extends to relational investment behaviors at the social level. Given these considerations, the present study has chosen to adopt the concept of parental investment behavior as the focus of investigation.

### 2.3 L2 learning motivation and parental involvement

While limited research has been conducted on parental investment behavior in language education, existing studies in general education and educational psychology provide valuable insights into the role of parental involvement. The present study reviewed the relevant studies on parental involvement in general education and educational psychology, from which the research in language education also gained affordance and insights. Positive parental support and investment have been shown to enhance students’ learning motivation, attitudes, and behaviors (e.g., Epstein et al., 2002; Pomerantz et al., 2007; Fan and Williams, 2010; Kong and Wang, 2021; Zhu et al., 2022; Wang and Liu, 2024). In a review of prior literature, Gonzalez-DeHass et al. (2005) concluded that parental engagement and participation positively impact students’ motivational constructs, such as self-regulation, intrinsic and extrinsic motivation, and mastery goals.

Fan and Williams (2010) found that four parental involvement variables (i.e., parental guidance, home-school contact, aspirations for tenth-grade students’ postsecondary education, and family rules concerning television watching) positively affected students’ intrinsic English motivation. The research results of Villiger et al. (2014) showed that parents’ emotional support predicted fourth-grade students’ reading enjoyment and curiosity, and parental expectations also impacted students’ reading curiosity. Kong and Wang (2021) demonstrated that parents’ support and perception of usefulness are positively correlated with children’s flow experience and intrinsic motivation in programming education. Boonk et al. (2022) concluded that parental involvement variables (parent–child discussions about education and parents’ expectations) had a positive impact on children’s motivation. Zhu et al. (2022) highlighted the importance of parental support in young L2 learners’ learning, suggesting that parents should spend time reading with their children to increase the learners’ motivation, learning engagement, and academic performance.

Based on the structural equation model, Liu (2017) identified a linear relation between parental investment belief, parental investment behavior, and middle school students’ English learning motivation. Specifically, parents’ investment behavior directly impacted students’ English learning motivation. Additionally, students’ motivation to study abroad was positively correlated with parental economic investment behavior, suggesting that more economic capital invested by parents would increase the opportunities for students to study abroad, as well as motivate them to do so. A further study conducted by Liu (2024) validated the internal structure of parental investment and found that parental investment belief had an indirect influence on high school students’ L2MSS mediated by parental investment behavior.

In general education, there has been extensive research on the impact of parental investment and involvement on students’ learning outcomes. However, there is limited research on the role of parental investment in learners’ language learning motivation. The family environment, serving as an immediate environment for students, exerts a substantial impact on students’ motivation to learn a foreign language (Gonzalez-DeHass et al., 2005; Schunk et al., 2014; Gong et al., 2023). Students’ English learning is supported by parental investment, facilitated through close communication within the family environment. The students’ motivation to learn English is influenced by both parental investment and interactions between students and their parents. This study aims to examine the relationship between different components of students’ L2 Motivational Self System (L2MSS) and various aspects of parental investment behavior to further explore the critical role of parents in the family context during the process of students’ English learning. Consequently, this study seeks to address three research questions.

*RQ1*: What are the levels of high school students’ L2MSS in terms of the ideal L2 self, the ought-to L2 self and the L2 learning experience?

*RQ2*: What are the levels of the parental investment behavior in terms of the economic, relationship, knowledge and emotional investment behavior?

*RQ3*: What is the relationship between students’ L2MSS and the parental investment behavior?

## 3 Methodology

### 3.1 Research contexts and participants

In China, English is a mandatory subject in high schools and is a significant component of the college entrance examination (Gao, 2014; Zhang and Liu, 2022). Parents play a crucial role in their children’s education, often actively engaging in their academic journey and aspiring for them to achieve high exam scores and improved career prospects (Liu, 2017, 2024). Given this context, this study selected high school students as its research participants. The convenience sampling method was utilized to survey Chinese high school students due to the accessibility it offered (Rose et al., 2019). A total of 900 students from two northeastern cities in China were involved in this study, consisting of 403 male students (44.8%) and 497 female students (55.2%).

### 3.2 Research instrument

The study utilized a composite questionnaire including the demographic information, the L2MSS Scale, and the Parental Investment Behavior Scale. All items employed the Likert 5-point scale ranging from “1 (Strongly Disagree)” to “5 (Strongly Agree).” Demographic information collected the students’ age, gender, and grade level as well as their parents’ educational level and social status.

The L2MSS Scale was based on the L2MSS part of *English Learner Questionnaire* by You and Dörnyei (2016), including 16 items distributed in three dimensions, namely, ideal L2 self (e.g., Q2: I can imagine myself in the future giving an English speech successfully to the public in the future.), ought-to L2 self (e.g., Q7: Studying English is important to me in order to gain the approval of the society.), and L2 learning experience (e.g., Q15: I think time passes faster while studying English.). The scale demonstrated high reliability, with global and dimensional Cronbach’s alpha coefficients of 0.885, 0.702, 0.684, and 0.760, respectively. The model’s fit met the ideal criteria set by Hair et al. (2019): χ^2^/df = 3.942 (<5), CFI (Comparative Fit Index) = 0.979 (>0.90), TLI (Tucker-Lewis Index) = 0.967 (>0.90), RMSEA (Root Mean Square Error of Approximation) = 0.057 (<0.08), and SRMR (Standardized Root Mean Square Residual) = 0.033 (<0.10).

The Parental Investment Behavior Scale was taken from the *Family Education and English Learning Questionnaire* designed and validated by Liu (2024), consisting of 14 items classified into four sub-dimensions of parental investment behavior, namely, economic investment behavior (e.g., Q17: My parents pay for my English tutoring classes), relationship investment behavior (e.g., Q22: My parents made an effort to find a way to enroll me in the class I am currently attending), knowledge investment behavior (e.g., Q23: My parents converse with me in English as a means of improving my language skills), and emotional investment behavior (e.g., Q18: My parents encourage me when I make progress in learning English). This scale demonstrated higher reliability, with Cronbach’s alpha coefficients for the four dimensions measuring 0.615, 0.803, 0.844, and 0.774, respectively. While the overall scale had a reliability of 0.847. The model fits meet the ideal criteria (Hair et al., 2019): χ^2^/df = 4.428 < 5; CFI = 0.948 > 0.90, TLI = 0.931 > 0.90; RMSEA = 0.062 < 0.008, and SRMR = 0.053 < 0.10.

### 3.3 Data collection and analysis

The questionnaire was administered in Chinese to facilitate accurate comprehension and appropriate responses from the students. Prior to commencing the survey, participants were provided with a thorough explanation of the survey’s purpose, and informed consents were obtained from both students and their teachers. A total of 900 valid questionnaires were collected. Data were subsequently entered and analyzed using SPSS version 24.0. First, descriptive statistics were calculated for both the L2MSS and the parental investment behavior, including maximum, minimum, mean, and standard deviation values. Subsequently, the Pearson correlation coefficient was computed to examine the relationship between students’ L2MSS and the parental investment behavior in response to the third research question. Lastly, a linear regression analysis was conducted to explore the relationship between the parental investment behavior and students’ L2MSS. This analysis utilized the three dimensions of L2MSS as dependent variables and the four dimensions of the parental investment behavior as independent variables.

## 4 Results

### 4.1 Profiles of the L2MSS and parental investment behavior

As shown in Table 1, the global L2MSS and its three components received ratings that were marginally above the midpoint level of 3, with the L2 learning experiences domain achieving the highest mean score (*M* = 3.33, *SD* = 0.84). Repeated measures ANOVA indicated significant variations in mean scores across the dimensions of the L2MSS: *F* (2, 1798) = 19.733, *p* < 0.001, partial η^2^ = 0.022. Specifically, pairwise comparisons with a Bonferroni adjusted alpha demonstrated that ideal L2 self (*M* = 3.16, *SD* = 0.82) achieved lower scores than L2 learning experience (*M* = 3.33, *SD* = 0.84) and ought-to L2 self (*M* = 3.31, *SD* = 0.85). No statistically significant difference was observed between the mean scores of L2 learning experiences and ought-to L2 self.

**Table 1:** Descriptive analyses of variables.

Dimension	Max	Min	M	SD
Ideal L2 self	5.00	1.00	3.16	0.82
Ought-to L2 self	5.00	1.00	3.31	0.85
L2 learning experience	5.00	1.00	3.33	0.84
L2 Motivational Self System	5.00	1.19	3.26	0.66
Economic investment	5.00	1.00	2.08	0.96
Relationship investment	5.00	1.00	1.96	1.08
Knowledge investment	5.00	1.00	1.44	0.70
Emotional investment	5.00	1.00	2.52	0.90
Parental investment behavior	4.88	1.00	2.00	0.66

The level of parental investment behavior in high school students was 2.00 (*SD* = 0.66), below average. In terms of parental economic investment behavior, the level was 2.08 (*SD* = 0.96). Two related items (i.e., paying for English tutoring classes and arranging for one-on-one English tutoring) had the mean values of 2.42 and 1.75. This study found that the level of parental relationship investment behavior was 1.96 (*SD* = 1.08). The mean values of three related items (i.e., finding a way to enroll in the current class, in the current school, and a conductive English learning environment) were 1.96, 2.06, and 1.85. The mean of parental knowledge investment behavior in this study was 1.44 (*SD* = 0.70). The mean values of the four related items (i.e., conversing in English, telling English stories, teaching English songs, teaching English knowledge) were 1.43, 1.44, 1.45, and 1.44. According to this study, the level of parental emotional investment behavior was 2.52 (*SD* = 0.90). As for the five related items (encouragement; helping analyze reasons about falling behind; setting an example by highlighting successful learners or by highlighting classmates; engagement in children’s English learning), the mean values were 3.18, 2.51, 2.14, 2.45, and 2.32.

### 4.2 Relationship between the L2MSS and parental investment behavior

Table 2 presented the results of Pearson correlation between students’ the L2MSS and parental investment behavior. There was a significant low correlation between the students’ L2MSS and the parental investment behavior (*r* = 0.170, *p* < 0.05) (see in Table 2). The results showed no significant correlation between students’ L2MSS and parental economic investment behavior. However, a significant low positive correlation existed between students’ L2MSS and parental relationship investment behavior (*r* = 0.262, *p* < 0.05). Students’ L2MSS was also positively correlated with parental knowledge investment behavior (*r* = 0.132, *p* < 0.05). Students’ L2MSS and parental emotional investment behavior exhibited a significant low positive correlation (r = 0.262, *p* < 0.05).

**Table 2:** Correlation coefficients of variables.

	Glboal PIBeH	EcoInBe	EmoInBe	RelationInBe	KnowInBe
Global L2MSS	0.170**	0.004	0.262**	0.262**	0.132**
Ideal L2 Self	0.188**	0.033	0.244**	0.158**	0.106**
Ought-L2Self	0.102**	−0.027	0.184**	0.034	0.133**
L2 learning experience	0.116**	0.006	0.197**	0.066*	0.074*

*N* = 900, ** *p* < 0.05, * *p* < 0.1.

L2MSS, L2 Motivational Self System; PIBeH, parental investment behavior; EcoInBe, economic investment behavior; RelationInBe, relationship investment behavior; KnowInBe, knowledge investment behavior; EmoInBe, emotional investment behavior.

Multiple linear regression was conducted to determine the best linear combination of the economic investment, the relationship investment, the knowledge investment and the emotional investment for predicting students’ L2MSS. Statistical assumptions, such as the normal distribution of residuals and the non-linear correlation between predicted variables and residuals were all met in the analysis. The means, standard deviations, and correlation coefficients could be found in Tables 1, 2. The regression method of “enter” showed that the combination of the four independent variables significantly predicted students’ L2MSS, *F* (4, 895) = 18.638, *p* < 0.01, with the economic investment and the emotional investment significantly contributing to the prediction (*p* < 0.05) except the relationship investment and the knowledge investment (*p* > 0.05) (see Table 3).

**Table 3:** Baseline regression analysis.

	Model (1)		Model (2)		Model (3)		Model (4)	
	L2MSS		IdealL2Self		OughtL2Self		L2LearnExp	
PIBeH	0.170***		0.188***		0.102**		0.116***	
EcoInBe		−0.106**		−0.078*		−0.105**		−0.069
EmoInBe		0.277***		0.236***		0.193***		0.219***
RelationInBe		0.021		0.098**		−0.037		0.008
KnowInBe		0.034		−0.010		0.092*		−0.004
_cons	2.923***	2.835***	2.691***	2.630***	3.047***	2.942***	3.029***	2.934***
*N*	900	900	900	900	900	900	900	900
*R* ^2^	0.029	0.078	0.035	0.069	0.010	0.049	0.014	0.043

*** *p* < 0.01, ** *p* < 0.05, * *p* < 0.1.

L2MSS, L2 Motivational Self System; IdealL2Self, ideal L2 self; OughtL2Self, ought-to L2 self; L2LearnExp, L2 learning experience; PIBeH, parental investment behavior; EcoInBe, economic investment behavior; RelationInBe, relationship investment behavior; KnowInBe, knowledge investment behavior; EmoInBe, emotional investment behavior.

Models 2 to 4 in Table 3 examined the effects of the parental investment behavior on the three sub-dimensions of students’ L2MSS. The results showed that the parental investment behavior had a positive effect on the three sub-dimensions of the L2MSS. Similarly, when the parental investment behavior was divided into four sub-dimensions for analysis on each sub-dimension of L2MSS, the economic investment behavior predicted students’ ideal L2 self and ought-to L2 self. The emotional investment behavior significantly predicted the three dimensions of students’ L2MSS. However, the relationship investment behavior only had a positive effect on the ideal L2 self, and the knowledge investment behavior had an effect on the ought-to L2 self.

## 5 Discussion

### 5.1 Profiles of the L2MSS and parental investment behavior

This study found that high school students had a higher level of the L2MSS, suggesting that the majority of students held a positive attitude towards learning English and maintained an ideal self-image related to language acquisition (You and Dörnyei, 2016; Lee and Lee, 2020; Thorsen et al., 2020). The levels of students’ ideal L2 self, ought-to L2 self and L2 learning experience were slightly higher than the average. The level of students’ L2 learning experience might relate to the learning atmosphere and teacher-student relationship (Liu, 2024). Students with a strong proficiency in English were more likely to receive recognition and support from peers and teachers, thereby achieving higher social standing in class and experiencing a more rewarding learning environment (Ryan, 2009; Zhang and Liu, 2022). Moreover, the high level of students’ ought-to L2 self could be explained that parents emphasized English as more than just an academic subject, positioning it as a key factor in their children’s future career prospects (Taguchi et al., 2009). High school students demonstrated motivation primarily driven by the desire to meet the expectations or gain recognition from parents, teachers, and peers (Lee and Lee, 2020; Zhang and Liu, 2022). Therefore, high school students’ primary focus was on achieving higher marks rather than envisioning English in future career contexts, which might result in a less defined ideal L2 self-image (Magid, 2009).

This study found that the level of the parental investment behavior in high school students was below average, indicating that parents may not fully recognize the significance of English and consequently they may not invest sufficient capital in their children’s English education. The parental investment and involvement could decrease as students grow older and the growing need of autonomy would reduce students’ dependence on parents (Boonk et al., 2022). The level of the parental economic investment behavior was below average which was in line with the findings of Liu (2024). The parental economic investment behavior is influenced by their investment beliefs and the economic resources available to them (Liu, 2017, 2024).

The study also indicated that the level of parental relationship investment behavior was below average, paralleling the findings of Liu (2024). This may be attributed to the relatively stable nature of school and class assignments, which reduces the likelihood of school or class changes. It might be difficult for teachers who were faced with the management and guidance of a large number of students to sustain a productive relationship with each student’s parents (Hill and Tyson, 2009).

The level of parental knowledge investment behavior was below average which might be influenced by parents’ educational level (Alawawda and Razi, 2020; Liu, 2024). Parents with lower educational level may be unable to offer sufficient English knowledge tutoring to their children (Fan and Chen, 2001). In most cases, parents can only assist their children in checking the completion of homework as the English knowledge become more complicated in high school.

Moreover, the study noted that parental emotional investment behavior was also below average. It might relate to high school students’ psychological characteristics. High school students had an increasing demand for independence from their parents and may not communicate with their parents frequently (Boonk et al., 2022). Additionally, some parents may not be able to dedicate time to accompanying their children in learning due to the challenge of balancing family economic responsibilities and their involvement in their children’s education (Hall and Quinn, 2014).

### 5.2 Relationship between the L2MSS and parental investment behavior

Parental investment and support serve as one of the most important factors for students’ English learning motivation (Gonzalez-DeHass et al., 2005; Schunk et al., 2014; Liu, 2024). The L2MSS is a habitus shaped within the sociolinguistic context and influenced by parents’ cultural, relationship, knowledge, and emotional capital (Liu, 2024). The study aimed to investigate the potential predictive relationship between different dimensions of parental investment behavior—namely, economic, relationship, knowledge, and emotional investment—and students’ L2MSS.

The results found in this study a significant low positive correlation between students’ L2MSS and parental investment behavior. This relationship suggests that students who benefit from higher levels of parental investment behavior may exhibit more pronounced L2MSS, and that increased student motivation could prompt greater parental investment in their children’s English education. The results were similar to the results reported by Liu (2024) who found that the parental investment behavior had a direct influence on students’ L2 learning motivation. The study’s findings underscore the critical role of parental support and investment in augmenting students’ motivation to learn English (Fan and Williams, 2010; Schunk et al., 2014; Boonk et al., 2022). By acknowledging the influence of parental investment on students’ language motivation, this research contributes to the broader understanding of the interplay between family dynamics and educational outcomes.

The results demonstrated no correlation between students’ L2MSS and economic investment behavior which was in line with the research findings of Liu (2024). There was a significant low positive correlation between students’ L2MSS and the relationship investment behavior, indicating that students whose parents offered more relationship investment might exhibit higher levels of the ideal L2 self, the ought-to L2 self, and the L2 learning experience. Parents’ active participation in school activities, such as attending parent meetings, may foster students’ academic participation to a certain extent and make students more motivated to engage in learning (Hill and Tyson, 2009). Parents’ participation in educational activities was the cornerstone of students’ success in school, attracting students’ interest in engaging in school activities (He et al., 2015).

This study identified a significant low positive correlation between L2MSS and the knowledge investment behavior. It indicated that students who received sufficient knowledge investment from their parents might develop a more specific image of their ideal L2 self and ought-to L2 self or obtain a greater L2 learning experience. Parents’ guidance in English knowledge learning could enhance students’ motivation to learn English (Hosseinpour et al., 2015; Alawawda and Razi, 2020). The parental knowledge investment behavior was mainly related to the cultural capital (Bourdieu, 1986) invested by parents in educating their children affected by their education level and background. Parents with higher education levels could be more aware of the vital role of mastering a foreign language in their children’s academic and future career development (Liu, 2024). The study identified a significant low positive relationship between students’ L2MSS and the emotional investment behavior, suggesting that with the continuous increase of parental emotional engagement, students might have higher levels of the three dimensions of L2MSS. Parents’ emotional encouragement and support helped students to form a positive evaluation of themselves. Even in the face of difficulties, students with enough emotional support could be motivated and confident to devote themselves to learning (Gonzalez-DeHass et al., 2005; He et al., 2015).

Multiple regression analyses indicated that parental investment behavior explained 7.8% of students’ L2MSS. It could be explained that students’ motivation to learn English was affected by various factors, including not only parents’ investment and involvement (Gonzalez-DeHass et al., 2005; Schunk et al., 2014; Liu, 2024), but also students’ internal factors (e.g., anxiety, personality, learning strategies or styles) (Papi, 2010; Dörnyei and Ryan, 2015) and other social factors, such as peers and teachers in the school, and other important people in the community (Fan and Williams, 2010; Schunk et al., 2014). The parental economic investment behavior was found to significantly predict students’ L2MSS, indicating that the more economic capital parents invest, the lower students’ English learning motivation. One possible reason might be that high school students become more independent psychologically and emotionally from parents. They may have heavy schoolwork, so the excessive parental economic investment in extracurricular classes might be seen as controlling and negatively influence students’ motivation (Fan and Williams, 2010). Parental emotional investment behavior emerged as a significant positive predictor of students’ L2MSS, which indicated that students with a higher level of parental emotional investment behavior had a higher level of L2MSS. Students whose parents affirm their worth and provide emotional support are more likely to attain their ideal self-images and have more confidence to avoid negative outcomes. When parents invest in students’ education such as setting an example and providing guidance, they can act as the capital which could help students encounter challenges and reinforce the value of learning (Gonzalez-DeHass et al., 2005).

## 6 Conclusion and implications

The present study investigated the relationship between high school students’ L2MSS and parental investment behavior. The results showed that the level of high school students’ L2MSS was slightly higher than the average and the level of parental investment behavior was below average. Students’ L2MSS is greatly affected by students’ psychological characteristics, parents’ expectations, and school requirements. The level of the parental emotional investment behavior was the highest while the parental knowledge investment behavior was the lowest. The parental investment behavior was influenced by factors such as the socioeconomic status of the parents, their educational level, and the amount of time they have available between work and tutoring their children at home. The present study identified a significant low positive correlation between students’ L2MSS and the parental investment behavior. The students with a higher level of parental investment behavior are likely more motivated to learn English. The increase in students’ English learning motivation may also promote more parental investment.

The present study implies the significance of parental emotional investment behavior such as encouraging students when they make progress, communicating frequently with students about their English learning as well as setting English learning role models. When parents are invested, students reported more motivation and take responsibility in learning English. Moreover, as significant others in the family environment, parents should create a comfortable and caring learning atmosphere and provide emotional support and encouragement for their children, taking into account adolescents’ psychological characteristics without focusing on peer comparisons. Parents might be more involved when they know their children are motivated. The findings provide insights for parents in which the four types of parental investment behavior do not play the same role in students’ English learning motivation. Parents could compensate for the disadvantage in financial support through adequate emotional investment for their children.

## 7 Limitations and recommendations

The present study was limited by the following aspects. In regards to research methods, this study was limited to obtaining data through questionnaires. To provide a comprehensive analysis and understanding of how parental investment behavior impacts students’ motivation to learn English, it would be beneficial to conduct further research using a variety of data collection methods such as observations, student journals, and interviews with parents and teachers (Rose et al., 2019). Furthermore, due to the availability of data, this study investigated only parental investment behavior and students’ L2MSS through a cross-sectional design. Future studies are advised to measure students’ motivation and the parental investment behavior over time and track changes in a longitudinal way. Additionally, the differences between various contexts, such as domestic or foreign educational contexts and urban or rural areas, should also be considered in the future research on parental investment.

## Data availability statement

The data analyzed in this study is subject to the following licenses/restrictions: further inquiries can be directed to the first author. Requests to access these datasets should be directed to zhangy435@nenu.edu.cn.

## Ethics statement

The studies involving humans were approved by Department of Applied Foreign Language Studies, Nanjing University. The studies were conducted in accordance with the local legislation and institutional requirements. Written informed consent for participation in this study was provided by the participants' legal guardians/next of kin.

## Author contributions

YZ: Conceptualization, Data curation, Formal analysis, Investigation, Methodology, Software, Writing – original draft, Writing – review & editing, Funding acquisition. XL: Conceptualization, Data curation, Methodology, Writing – original draft, Writing – review & editing, Investigation. HL: Conceptualization, Data curation, Funding acquisition, Investigation, Supervision, Writing – original draft, Writing – review & editing.
